# Is selenoprotein K required for *Borrelia burgdorferi* infection within the tick vector *Ixodes scapularis*?

**DOI:** 10.1186/s13071-019-3548-y

**Published:** 2019-06-07

**Authors:** Deepak Kumar, Monica Embers, Thomas N. Mather, Shahid Karim

**Affiliations:** 1 Department of of Cell and Molecular Biology, School of Biological, Environmental, and Earth Sciences, The University of Southern Mississippi, Hattiesburg, MS 39406 USA; 2 Division of Immunology, Tulane National Primate Research Center, Covington, LA 70455 USA; 3 Center for Vector-Borne Disease, University of Rhode Island, Kingston, RI 02881 USA

**Keywords:** Tick, *Ixodes scapularis*, *Borrelia burgdorferi*, Lyme disease, Selenoproteins, ER stress

## Abstract

**Background:**

Tick selenoproteins are involved in regulating oxidative and endoplasmic reticulum stress during prolonged tick feeding on mammalian hosts. How selenoproteins are activated upon tick-borne pathogen infection is yet to be defined.

**Methods:**

To examine the functional role of selenoprotein K in *Borrelia burgdorferi* infection within the tick host *Ixodes scapularis*, RNA interference (RNAi)-based gene silencing was performed.

**Results:**

Selenoprotein K is an endoplasmic reticulum (ER)-resident protein and a component of the ERAD complex involved in ER homeostasis. A qRT-PCR assay revealed the significant upregulation of *selenogene K* (*selenoK*) expression in *B. burgdorferi*-infected tick tissues. Silencing of the *selenoK* transcript significantly depleted *B. burgdorferi* copies within the infected tick tissues. Upon *selenoK* knockdown, another component of the ERAD complex, selenoprotein S (*selenoS*), was significantly upregulated, suggesting a compensatory mechanism to maintain ER homeostasis within the tick tissues. Knockdown of selenoK also upregulated ER stress-related unfolded protein response (UPR) pathway components, *ATF6* and *EIF2*.

**Conclusions:**

The exact mechanisms that contribute to depletion of *B. burgdorferi* upon *selenoK* knockdown is yet to be determined, but this study suggests that *selenoK* may play a vital role in the survival of *B. burgdorferi* within the tick host.

**Electronic supplementary material:**

The online version of this article (10.1186/s13071-019-3548-y) contains supplementary material, which is available to authorized users.

## Background

In the USA, reported vector-borne diseases are predominantly tick-borne; for example, there are approximately 329,000 cases of Lyme disease annually in the USA. A recent CDC study based upon vector-borne disease cases reported to the National Notifiable Disease Surveillance System from 2004 to 2016 revealed 491,671 cases of tick-borne infectious diseases (76.5% of all vector-borne diseases) (http://ww.cdc.gov/mmwr) [1]. In the USA, Lyme disease-causing *B. burgdorferi* spirochetes are primarily harbored by the blacklegged tick *I. scapularis* [2].

Tick blood-feeding also generates toxic levels of reactive oxygen species (ROS) that could damage lipids, proteins and DNA, and promote mutation, cellular dysfunction and cell death. To successfully feed and survive, ticks must somehow prevent these detrimental effects and promote the beneficial aspects of ROS, which suggests that there are precise regulatory strategies for maintaining appropriate ROS levels both within the tick and possibly at the tick-host interface. Our previously published studies have shown an adaptive coevolutionary process that has enabled tick-borne pathogen survival by manipulating an antioxidant defense system associated with selenium including a full set of selenoproteins and other antioxidants [3–12]. Generation of ROS is among the first lines of host defense against invading microbes [13, 14].

Selenoproteins exhibit diverse biological functions such as detoxification of peroxides, regeneration of reduced thioredoxin and reduction of oxidized methionine residues by oxidation of the selenium (Se^−^) active site [15–17]. In the last decade, significant progress has been made in clarifying the functions and physiological roles of vertebrate selenoproteins; new selenoprotein families have been identified, and new functions have been assigned to previously characterized selenoproteins. Some of the newer specific functions of selenoproteins involve removal of hydrogen peroxide, repair of oxidatively damaged proteins, control of cytoskeleton/actin assembly, protein folding and mitigating ER stress as part of the endoplasmic reticulum associated degradation (ERAD) complex [17], among others. Tick selenoproteins have been shown to play an important role in mitigating oxidative stress [3–12], pathogen colonization [5, 7–9, 11, 18–20] and microbiota maintenance [9, 10, 12].

A study using a tick-pathogen model has shown that the initial stage of transmission of a pathogen by an arthropod vector is influenced by gene expression changes in both vectors and pathogens [21]. However, there is a lack of knowledge on how gene expression within the vector is altered by the presence of the vector-borne pathogen. Therefore, the present study was designed to test the hypothesis that *B. burgdorferi* infection activates upregulation of selenogene K expression in order to survive within the tick host, *I. scapularis*, before transmission to the mammalian host. To test this hypothesis, a qRT-PCR assay was used to determine the expression of *selenoK* in *B. burgdorferi*-infected *I. scapularis* tissues over the course of a feeding period. An RNA interference approach also was used to silence selenogene K expression in *B. burgdorferi*-infected ticks to examine pathogen survival within the tick vector.

## Methods

### Materials

All common laboratory supplies and chemicals were purchased from Sigma-Aldrich (St. Louis, MO, USA), Fisher Scientific (Grand Island, NY, USA) or Bio-Rad (Hercules, CA, USA) unless otherwise specified.

### Bioinformatics analysis of selenoprotein K

The full-length gene sequence of *I. scapularis selenoK* (GenBank: XM_002403043.1) was obtained from the NCBI database. Corresponding protein sequences for *I. scapularis* selenoK (NCBI Accession number: XP_002403087.1) were aligned with selenoK protein sequences from other organisms such as *Amblyomma maculatum*, *A. americanum*, *Gallus gallus*, *Rattus norvegicus*, *Mus musculus*, *Equus caballus*, *Homo sapiens*, *Macaca mulatta*, *Pan troglodytes* and *Pongo abelii* by using Clustal Omega [22] for multiple sequence alignment. selenoK protein sequences aligned by Clustal Omega were refined by eye and presented by Jalview v.2.7 [23]. Additionally, the freely available online tool SECISsearch3 (http://seblastian.crg.es/) was used for the prediction of the SECIS (Selenocysteine Insertion Sequence) element in the tick *selenoK* nucleotide sequence, required for amino acid selenocysteine (Sec) insertion during translation [24]. Subcellular localization of tick *selenoK* was also predicted by using the freely available online algorithm DeepLoc-1.0 (http://www.cbs.dtu.dk/services/DeepLoc/index.php) which predicts the subcellular localization of eukaryotic proteins [25].

### Ticks and tissue dissections

Ticks were purchased from the Oklahoma State University Tick Rearing Facility. Adult male and female *I. scapularis* were kept according to standard practices [26] and maintained in the laboratory as described in our previously published work [12]. Unfed female adult *I. scapularis* were infected with laboratory grown *B. burgdorferi* strain B31.5A19 using the capillary feeding method at Tulane National Primate Research Center [27]. Naturally *B. burgdorferi-*infected adult *I. scapularis* were collected from field (Kingston, Rhode Island) and maintained in the laboratory using standard procedures [28]. Rabbit was used as host for tick blood-feeding. The blood-fed adult female *I. scapularis* were dissected within 60 min of removal and collection from the rabbit. Tick tissues were dissected and washed in M-199 buffer as described previously [12]. Salivary glands and midguts from individual *I. scapularis* were stored in RNAlater (Life Technologies, Carlsbad, CA, USA) at − 80 °C until used.

### Total RNA isolation, cDNA synthesis, dsRNA preparation and transcriptional expression

The methods to extract total RNA, cDNA synthesis, double-stranded RNA for selenoK, irrelevant gene LacZ, and qRT-PCR assays were performed as described previously [9, 12]. RNA was extracted from individually-dissected tick tissues (salivary gland, midgut), and cDNA was synthesized from each of them to check whether they were infected or not. Only tissues which were found infected were pursued for further work. The concentration of dsRNA used was 1000 ng/µl. One microliter of dsRNA was injected into the tick hemocoel. To investigate the role of selenoK in tick feeding success and pathogen survival, 45 unfed adult female capillary-fed (*B. burgdorferi* culture) ticks were injected with 1000 ng of selenoK-dsRNA into the hemocoel using a Hamilton syringe fitted with a 33-gauge needle [29]. As a control, a total of 45 unfed adult capillary-fed (*B. burgdorferi* culture) ticks were injected with 1000 ng of lacZ-dsRNA (an irrelevant control dsRNA). After the injection of dsRNA, ticks were kept at 37 °C overnight under high humidity to confirm tick survival. *Ixodes scapularis* ticks were then placed on rabbit ears [30]. *Ixodes scapularis* naturally infected with *B. burgdorferi* were collected from Rhode Island to determine the role of *selenoK* silencing on pathogen infection. A total of 90 female adult ticks were divided into two groups: one group of 45 female unfed ticks was injected with irrelevant dsRNA-lacZ; the second with selenoK dsRNA followed by tick infestation on rabbit ears as described earlier.

### PCR-based detection of *B. burgdorferi*

*Borrelia burgdorferi* was detected in the tick tissues using the *flaB* gene in a PCR assay [31]. The *flaB* gene and other primers used in the experiments are provided in Additional file 1: Table S1. The PCR conditions were followed from a previous study with slight modification [31]: 1 cycle of 94 °C for 5 min; 50 cycles of 94 °C for 30 s, 50 °C for 30 s and 68 °C for 1 min; and 1 cycle of 72 °C for 8 min.

### Quantification of *B. burgdorferi* in *I. scapularis* tissues

The principle of quantification of *B. burgdorferi* load was followed from our studies established to quantify the load of the spotted fever group rickettsia, *Rickettsia parkeri*, in tick tissues [9]. *Borrelia burgdorferi* load in *I. scapularis* tissues were estimated by quantifying the number of copies of *B. burgdorferi flagellin* gene, *flaB* [32] present per copy of housekeeping gene *Rps4* [30]. A list of all primers used is provided in Additional file 1: Table S1. The gene *flaB* from *B. burgdorferi* and *Rps4* from *I. scapularis* were amplified, purified, sequenced and verified prior to further assays. Their standard curves were determined by qRT-PCR based on serially-diluted PCR products. qRT-PCR conditions were as follows: 50 °C for 3 min, 95 °C for 10 min; followed by 40 cycles of 95 °C for 15 s, 60 °C for 30 s and 72 °C for 30 s.

### Statistical analysis

All data were expressed as mean values ± standard error of the mean (SEM) unless otherwise indicated. Statistical significance between two experimental groups or their respective controls was determined by a t-test (*P*-value, < 0.05). *P* < 0.05 was considered statistically significant. Comparative differences among multiple experimental groups were determined by analysis of variance with *P*-values < 0.05 considered statistically significant (GraphPad Prism 6.05; GraphPad Software, La Jolla, CA, USA). Transcriptional expression levels were determined using Bio-Rad CFX MANAGER v.3.1, and the gene expression values obtained were considered statistically significant if a *P*-value of 0.05 was obtained when compared with the control.

## Results and discussion

### Bioinformatics analysis

**Fig. 1:**
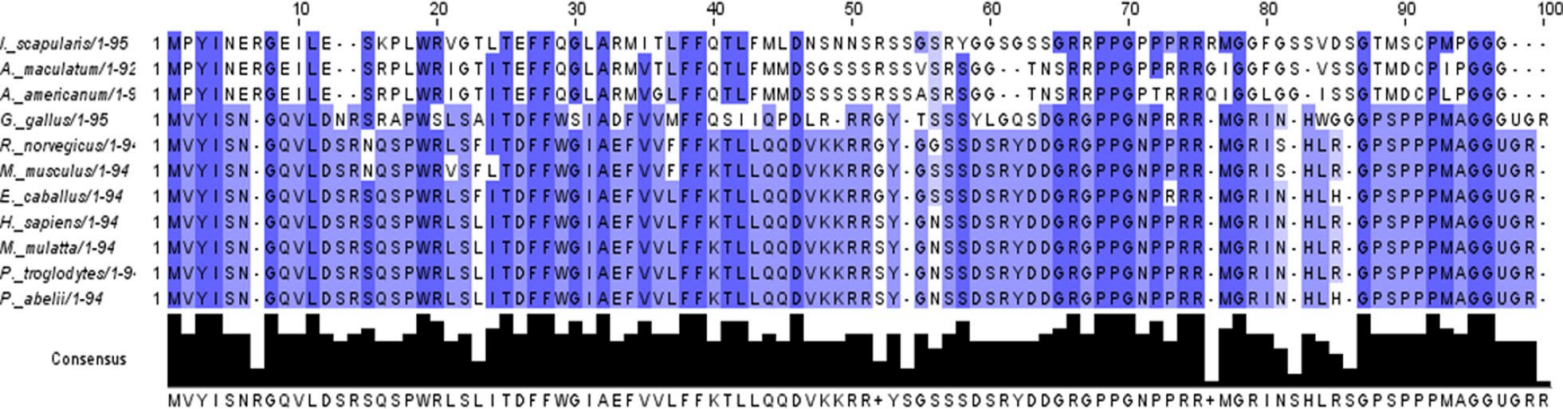
Multiple sequence alignment of selenoK. Tick *selenoK* sequences (as shown above, *I. scapularis*, *A. maculatum* and *A. americanum*) demonstrate 77% amino acid identity with each other but share only 38–40% amino acid identity with *Homo sapiens*

Selenoprotein K is a small (~ 10 kDa) and single spanning transmembrane protein localized on the ER membrane [33]. It contains the selenocysteine residue near the c-terminus in the cytosolic region, which remains in a highly disordered state [34]. *Ixodes scapularis*, *Amblyomma maculatum,* and *A. americanum selenoK* sequences (Fig. 1) demonstrate 77% amino acid identity with each other but share only 38–40% amino acid identity with *Homo sapiens*. Interestingly, many of the assembled transcripts in our unpublished RNA-Seq data are novel and not found in the *I. scapularis* annotation. This is not surprising, since the genome of *I. scapularis* has 50% coverage. Based on bioinformatics analysis, *I. scapularis* contains most of the selenogenes which have already been characterized in *A. maculatum* [5–7, 9, 10]. Interestingly, *I. scapularis selenoK* has 77% amino acid identity with that of *A. maculatum* suggesting conserved functions. In this work, the selenocysteine insertion element (SECIS) of tick *selenoK* was also predicted by algorithm SECISSearch3 [24]. The SECIS element is a hairpin loop structure required in a selenoprotein transcript for incorporation of selenocysteine (Sec) residue during translation. SECISSearch3 algorithm has been developed to detect SECIS elements in prospective selenogene sequences by using different parameters [24]. SECISSearch3 prediction is the combined output of three sources: infernal, used by the program cmsearch, SECIS model, used by the program Covels and the third source is the original program SECISSearch [35, 36]. Submission of *I. scapularis selenoK* sequence to the SECISSearch3 algorithm resulted into output data (Additional file 2: Figure S1). Generally, vertebrates have an infernal score of > 20 and a Covel score of > 15; in other species these scores are generally lower. The Covel score provides a quantitative measure of how better fit is the prediction to the SECIS model. An RNAfold algorithm is run to calculate the minimum free energy of SECIS structures among those suggested. Start to end positions of the SECIS element, and the strands on which it is located, is also given in the output data. The marker for SECIS element prediction quality is based on the experience of manual analysis of thousands of SECIS elements, and is graded as A, B or C in decreasing order of prediction quality. In our output data, infernal and Covel scores of the *selenoK* SECIS element were 19.62 and 21.35, respectively (Additional file 2: Figure S1). In our data, the grade provided by the algorithm to tick *selenoK* prediction was A, indicating the most trustworthy prediction possible under the algorithm parameters.

In the present work, subcellular localization of tick *selenoK* was predicted by DeepLoc-v.1.0 server which uses neural network algorithms based on the experimental localization of Uniprot proteins [25]. To include the protein sorting pathways into the algorithm, a hierarchical tree with multiple nodes was also designed. Each node of the tree represents a binary attempt to assign the protein right pathway from high to low in a hierarchical classification. As shown in Additional file 3: Figure S2, the probability score of tick *selenoK* is much higher at the ER/Golgi node than other organelles. Thus, our data suggest that the localization of tick *selenoK* is in the endoplasmic reticulum ER/Golgi membrane (Additional file 3: Figure S2). A previous study on *Drosophila* has also reported *selenoK* integration into both of these cell organelles [37]. Attention plot (Alpha) in Additional file 3: Figure S2 indicated that the specific interspersed region of the selenoprotein K (residue number ~ 20–40 from the N-terminal) contributes in binding to ER/Golgi membrane.

It has been suggested that all of the characterized selenoproteins can reduce ROS levels in the cell. However, at the physiological level, their specific roles are yet to be discovered in arthropod vectors like ticks. *selenoK* lacks the defined redox motif C-X-X-Sec (X = any amino acid) found in other known selenoproteins. Thus, at this time, it cannot be firmly stated that *selenoK* has an antioxidant role *in vivo*. It could be speculated that *selenoK* is part of the protein complex, which demonstrates its antioxidant property (Additional file 3: Figure S2). The exact cellular localization and function of tick selenoprotein in hematophagy and pathogen infection has yet to be determined.

### Temporal expression during ingestion of the blood meal

**Fig. 2:**
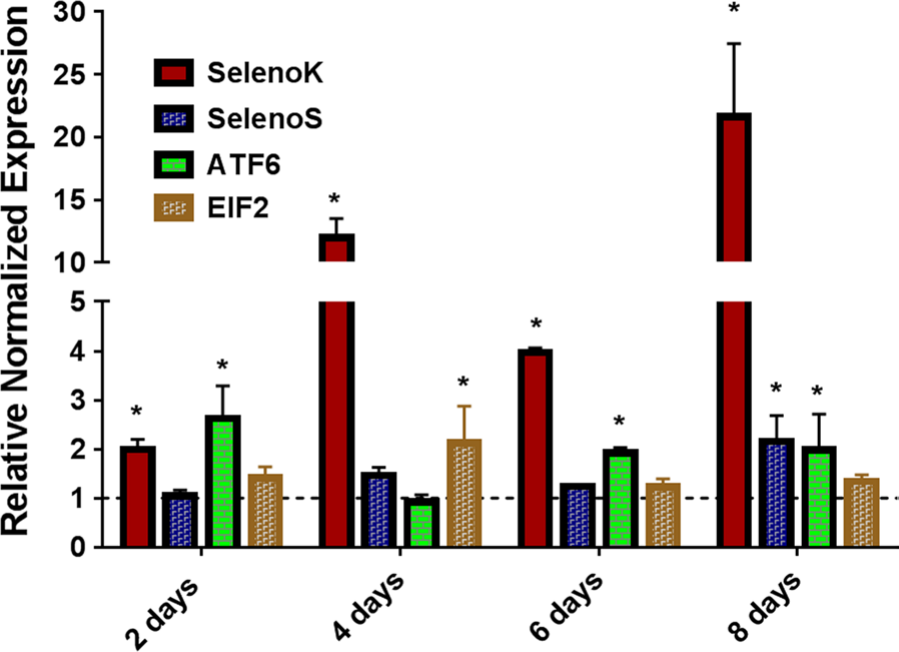
Temporal expression of ER-stress related genes in *Borrelia burgdorferi-*infected ticks. ATF6 and EIF2 are unfolded protein response related sensor genes while *selenoK* and *selenoS* are components of the ERAD complex involved in ER stress mitigation through the ERAD pathway. Gene expression of the mentioned genes was normalized with that of clean tick midgut gene expression (indicated as 1 on the y-axis). Statistically significant gene expression values (*P* < 0.05) are indicated with * (asterisk). Rps4 was used as a housekeeping gene. *Abbreviations*: ATF6, activating transcription factor 6; EIF2, eukaryotic initiation factor 2 (eIF2)

Our recently published work showed the pathogen-induced expression of several tick selenoproteins [7, 9]. Several studies implicated the involvement of *selenoK* in mitigating endoplasmic reticulum (ER) stress generated upon microbial infection and elevated oxidative stress [38–40]. These and bioinformatics results prompted us to determine the the functional role of *selenoK* in *B. burgdorferi* infection of the tick vector. The *B. burgdorferi* infection level of capillary-fed and naturally-infected *I. scapularis* was determined using *flaB* (*flagellin*) gene PCR. The *B. burgdorferi* infection level in capillary-fed ticks and field-collected ticks ranged between 10–20%. *Borrelia burgdorferi* multiplies in midgut tissues before trafficking to the salivary glands. Therefore, the time-dependent expression of ER-resident *selenoK*, *selenoS* and UPR pathway genes *ATF6* and *EIF2* were examined in *B. burgdorferi-*infected midgut tissues. Transcriptional expression of *selenoK* significantly increased from 2- to 20-fold in tick tissues from day 2 to day 8 of blood-feeding (2 days, *t* = 4.627, *P* = 0.0098; 4 days, *t* = 10.94, *P* = 0.0004; 6 days, *t* = 24.8, *P* < 0.0001; 8 days, *t* = 3.525, *P* = 0.0243) (Fig. 2) supporting its critical role in *B. burgdorferi* colonization of tick tissues. Other upregulated ER-stress related genes during blood-feeding are *selenoS* (8 days, *t* = 3.462, *P* = 0.0258), *ATF6* (2 days, *t* = 3.404, *P* = 0.0272; 6 days, *t* = 7.505, *P* = 0.0017; 8 days, *t* = 3.142, *P* = 0.0348), *EIF2* (4 days, *t* = 2.812, *P* = 0.0482). selenoK is an ER-resident selenoprotein and one of the constituents of the ERAD complex which has a role in mitigating ER stress [41, 42]. Previous studies have also revealed that ER stress increases during pathogen infection and elevated oxidative stress [38–40]. According to one estimate, 70–80% of oxidative stress in the cell is due to mitochondria and mitochondrial ROS, and pathogen infection can disturb the ER homeostasis by disrupting protein folding and ER calcium ion balance which may result in elevated ER stress levels [43]. A previous study has already shown a physical and biochemical interaction between the ER and mitochondria [43, 44]. Since *selenoK* is an ER-resident selenoprotein and is one of the constituents of the ERAD complex which has a role in mitigating ER stress [41, 42], the significant upregulation of *selenoK* provided the basis of suggesting a functional role of *selenoK* in *B. burgdorferi* colonization within the tick. Other ER-stress related genes considered in this study during blood-feeding were *SelS*, *ATF6* and *EIF2*. Unfolded protein response (UPR) sensor related genes *ATF6* and *EIF2* also demonstrated upregulation during blood-feeding.

### SelenoK knockdown and *B. burgdorferi* infection

**Fig. 3:**
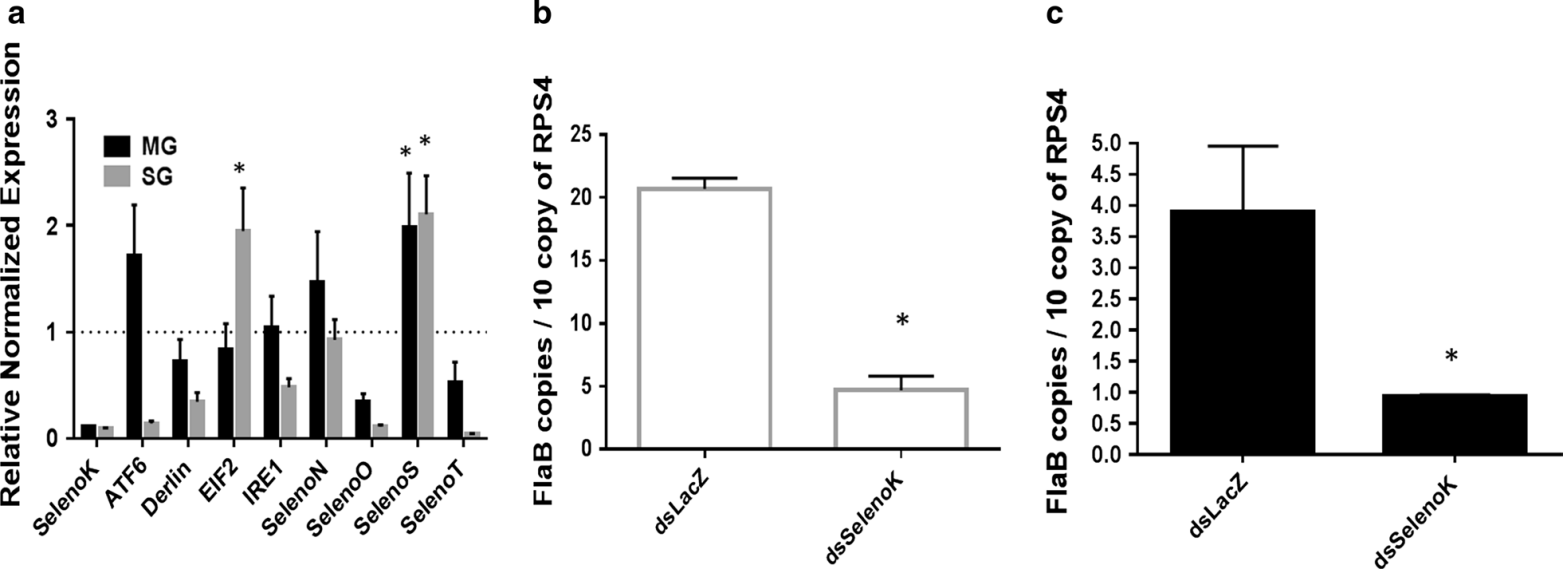
Effect of selenoK knockdown on *Borrelia burgdorferi* infection (capillary-fed induced infection). **a** Compensatory expression of ER stress genes when selenoK is knocked down in *B. burgdorferi*-infected tick tissues. *selenoS*, *ATF6* and *EIF2* demonstrate compensatory expression. selenoS and selenoK localized on ER membrane and are components of the ERAD, *selenoS* demonstrates compensation for *selenoK* in both tissues (SG, MG). **b**, **c** Impact of selenoK knockdown on *B*. *burgdorferi* infection in SG (**b**) and MG (**c**) tissues. Statistically significant gene expression values (*P* < 0.05) are indicated with * (asterisk). Rps4 was used as a housekeeping gene. *Abbreviations*: ATF6, activating transcription factor 6; EIF2, eukaryotic initiation factor 2; IRE1, inositol-requiring enzyme; Rps4, ribosomal protein S4; SG, salivary gland; MG, midgut

Injection of selenoK-dsRNA into *I. scapularis* female ticks resulted in an 80–90% reduction in corresponding transcript levels in the salivary gland (SG) and midgut (MG) tissues (Fig. 3a). One of the limitations of this study was that the *B. burgdorferi* infection rate was 10–20%, hence it was necessary to check every tick for infection and to quantify the infection level. In capillary-fed ticks, the infection level varied from tick to tick, whereas naturally-infected ticks had a more uniform level of infection. Interestingly, *selenoK* silencing significantly upregulated the expression of *selenoS*, suggesting a compensatory mechanism in this tick species, also shown in a recent study from our laboratory [9].

**Fig. 4:**
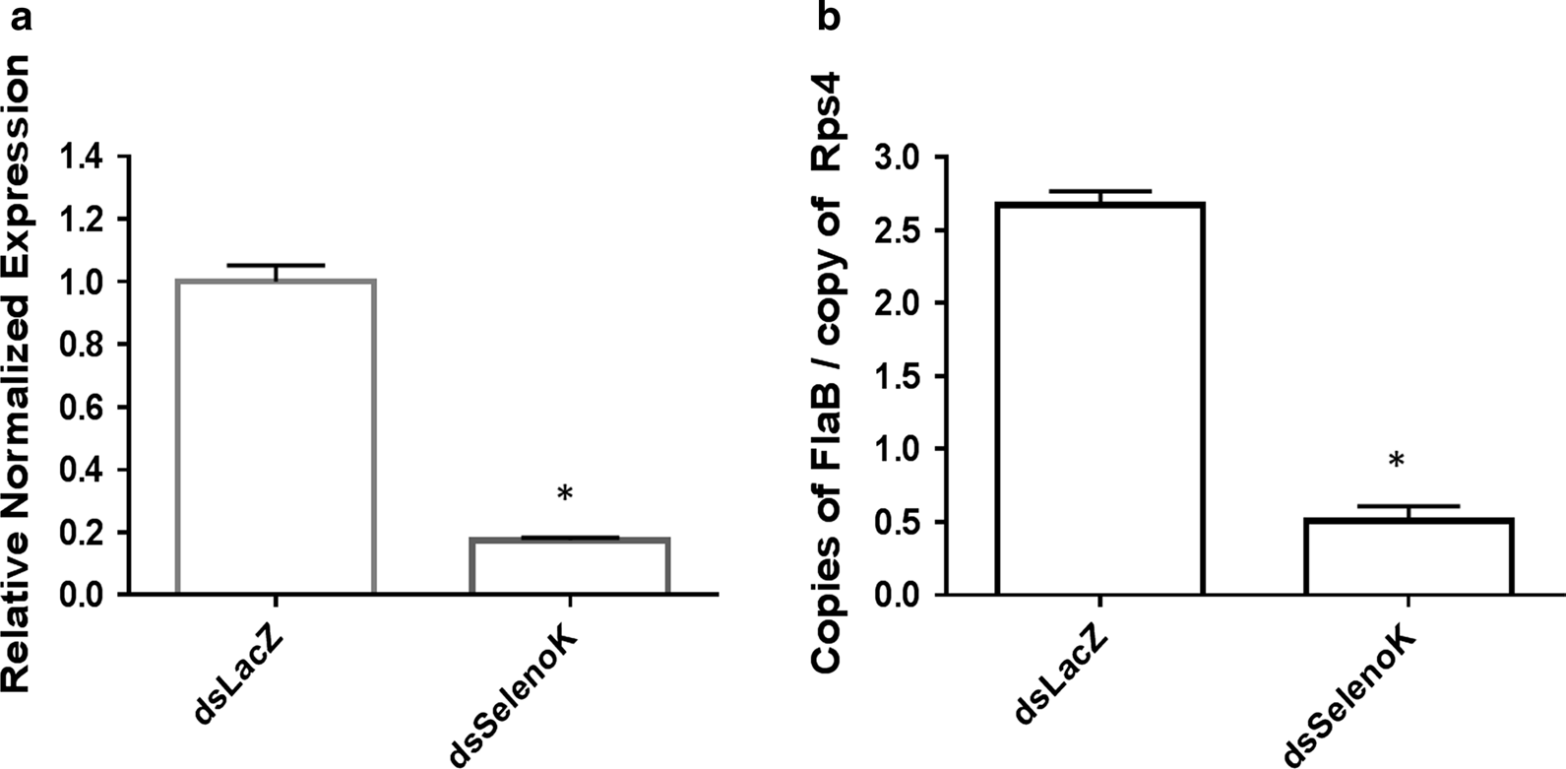
Effect of *selenoK* knockdown on *Borrelia burgdorferi* infection (naturally-infected ticks) **a** Knockdown level of *selenoK* in infected tick tissue. **b** Impact of *selenoK* knockdown on *Borrelia* load in ticks. Statistically significant gene expression values (*P* < 0.05) are indicated with * (asterisk). *Rps4* was used as a housekeeping gene

It has been reported that two cellular functions exist for *selenoK*, including ER stress mitigation by participation in the ER-associated protein degradation (ERAD) pathway, and regulation of Ca2+ flux from the ER [34]. Its speculative role in ERAD is based on the evidence that its transcripts were upregulated during ER stress [33], and later an ER stress response element (ERSE) was identified in the promoter of human *selenoK* [33]. Studies have demonstrated that *selenoK* is bound within the ERAD complex along with selenoS, p97 (VCP; valosin-containing protein) and the Derlins [33, 45]. In the present study, upregulation of *ATF6* and *EIF2* indicated an elevation in ER stress (Fig. 3a, for *EIF2*, *t* = 3.099, *P* = 0.0363), and upregulation of *selenoS* (~ 2 folds) in both SG and MG (Fig. 3a; for MG tissue, *t* = 3.143, *P* = 0.0347; for SG tissue, *t* = 3.055, *P* = 0.0379) indicate a partial compensatory mechanism (Fig. 3a). *Borrelia burgdorferi* load was significantly altered in *selenoK* knockdown salivary gland and midgut tissues (Fig. 3b, c; *F*_(3,8)_ = 158.7, *P* < 0.0001). One-way ANOVA using multiple comparison was used with Tukeyʼs test. *SelenoS* also is one of the components of the ERAD complex required to mitigate ER stress. Previous studies have shown that *selenoK* knockout mice appeared healthy, fertile and without any ER stress [33], probably because of redundancy or compensation [34]. Silencing of selenoK showed significant reduction in *B. burgdorferi* copies in both SG and MG tissues (Fig. 3b, c; for SG, *t* = 11.32, *P* = 0.0003; for MG, *t* = 4.508, *P* = 0.0108). The uneven infection level of *B. burgdorferi* in capillary-fed ticks prompted us to use naturally infected ticks to confirm these results (Fig. 4a, b). Upon selenoK knockdown in ticks, naturally infected *I. scapularis* ticks also demonstrated reduction in *B. burgdorferi* load (Fig. 4a, *selenoK* knockdown, *t* = 15.68, *P* < 0.0001; Fig. 4b, bacterial load reduction, *t* = 15.82, *P* < 0.0001).

## Conclusions

This proof-of-concept study suggests a role for the ER stress machinery in *B. burgdorferi* colonization and survival inside ticks. Our data provide a deeper insight into the possible role of *selenoK* in the pathogen colonization of tick vectors. The nymphal ticks play an epidemiologically important role in the infection of humans with *B. burgdorferi* and a detailed mechanistic study to investigate the role of ER-resident selenoproteins in pathogen infection and transmission is still needed.

## Additional files

**Additional file 1: Table S1.** Gene-specific PCR and qRT-PCR primers used in this study.

**Additional file 2: Figure S1.** SECIS prediction for *Ixodes scapularis* selenoK (XM_002403043.1) by SECISsearch3 algorithm. SECISsearch3 predicts the potential SECIS (selenocysteine insertion sequences) element for eukaryotes required for translation of selenoprotein from its mRNA.

**Additional file 3: Figure S2.** Prediction of subcellular localization of tick selenoprotein K (XP_002403087.1) by DeepLoc-1.0 algorithm which predicts its localization in the ER/Golgi membrane.

## Data Availability

The datasets supporting the conclusions of this article are included within the article and its additional files. Raw data are available from the corresponding author upon request.

## Abbreviations

ERAD: endoplasmic reticulum associated degradation
ATF6: activating transcription factor 6
EIF2: eukaryotic initiation factor 2
IRE1: inositol-requiring enzyme
Rps4: ribosomal protein S4
SG: salivary gland
MG: midgut
qRT-PCR: quantitative real-time polymerase chain reaction
ROS: reactive oxygen species
