# Supplementary figures and images for: Is selenoprotein K required for Borrelia burgdorferi infection within the tick vector Ixodes scapularis?
Source: Parasit Vectors. 2019 Jun 7;12:289. doi: 10.1186/s13071-019-3548-y (PMC6555942; doi:10.1186/s13071-019-3548-y)

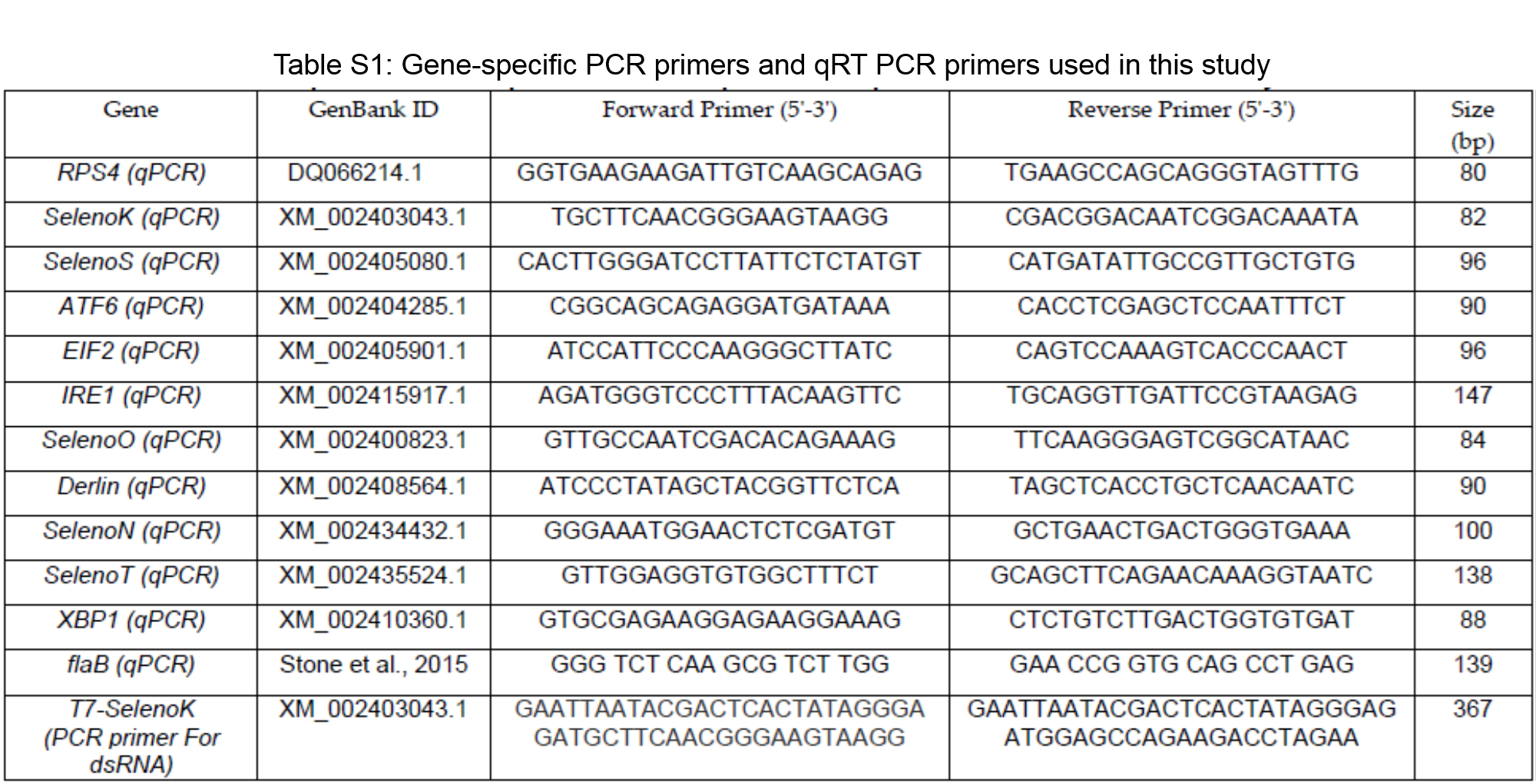

Supplement: Supplementary file 1 — Additional file 1: Table S1. Gene-specific PCR and qRT-PCR primers used in this study. [file 13071_2019_3548_MOESM1_ESM.tif]

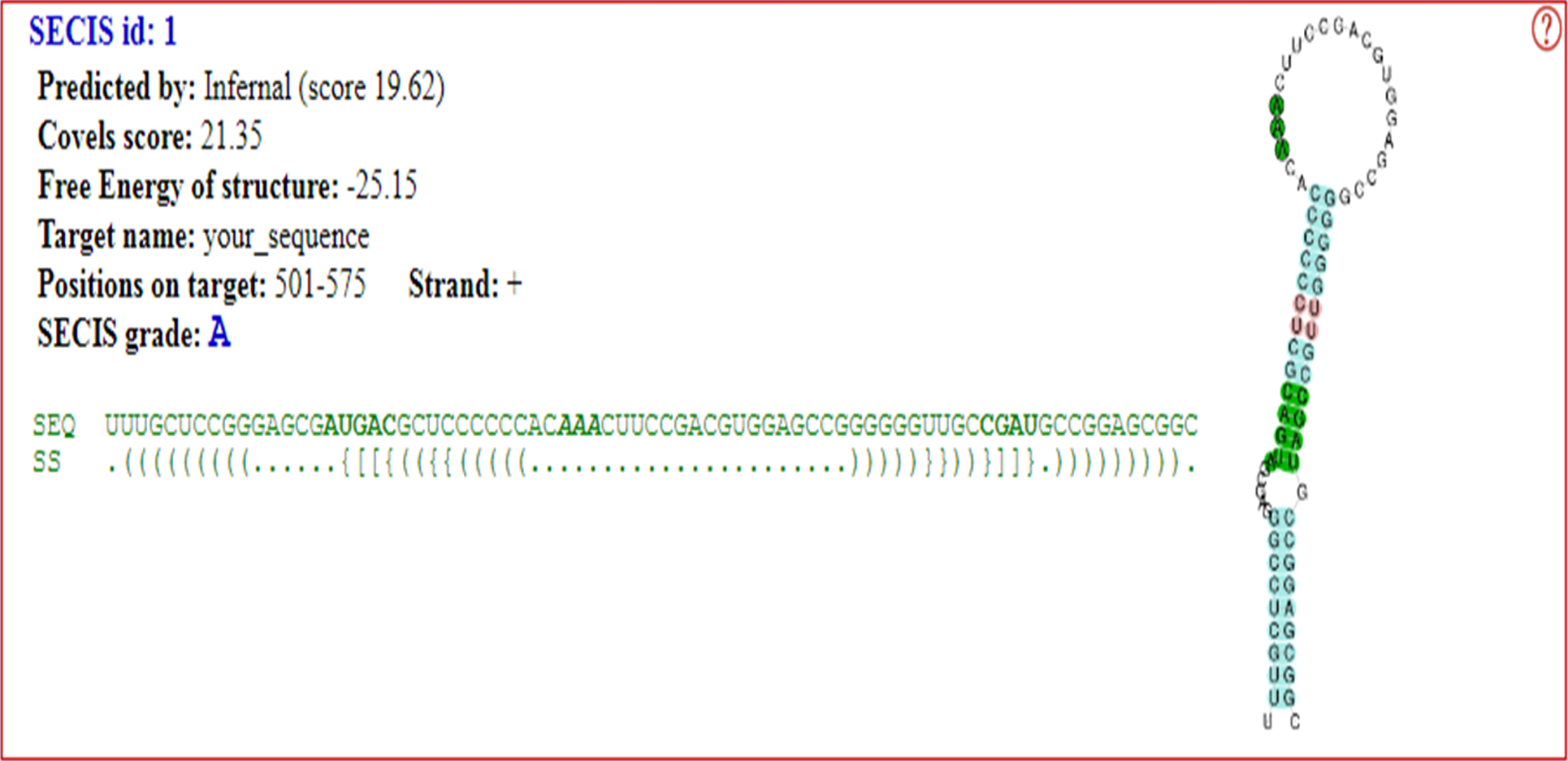

Supplement: Supplementary file 2 — Additional file 2: Figure S1. SECIS prediction for Ixodes scapularis selenoK (XM_002403043.1) by SECISsearch3 algorithm. SECISsearch3 predicts the potential SECIS (selenocysteine insertion sequences) element for eukaryotes required for translation of selenoprotein from its mRNA. [file 13071_2019_3548_MOESM2_ESM.tif]

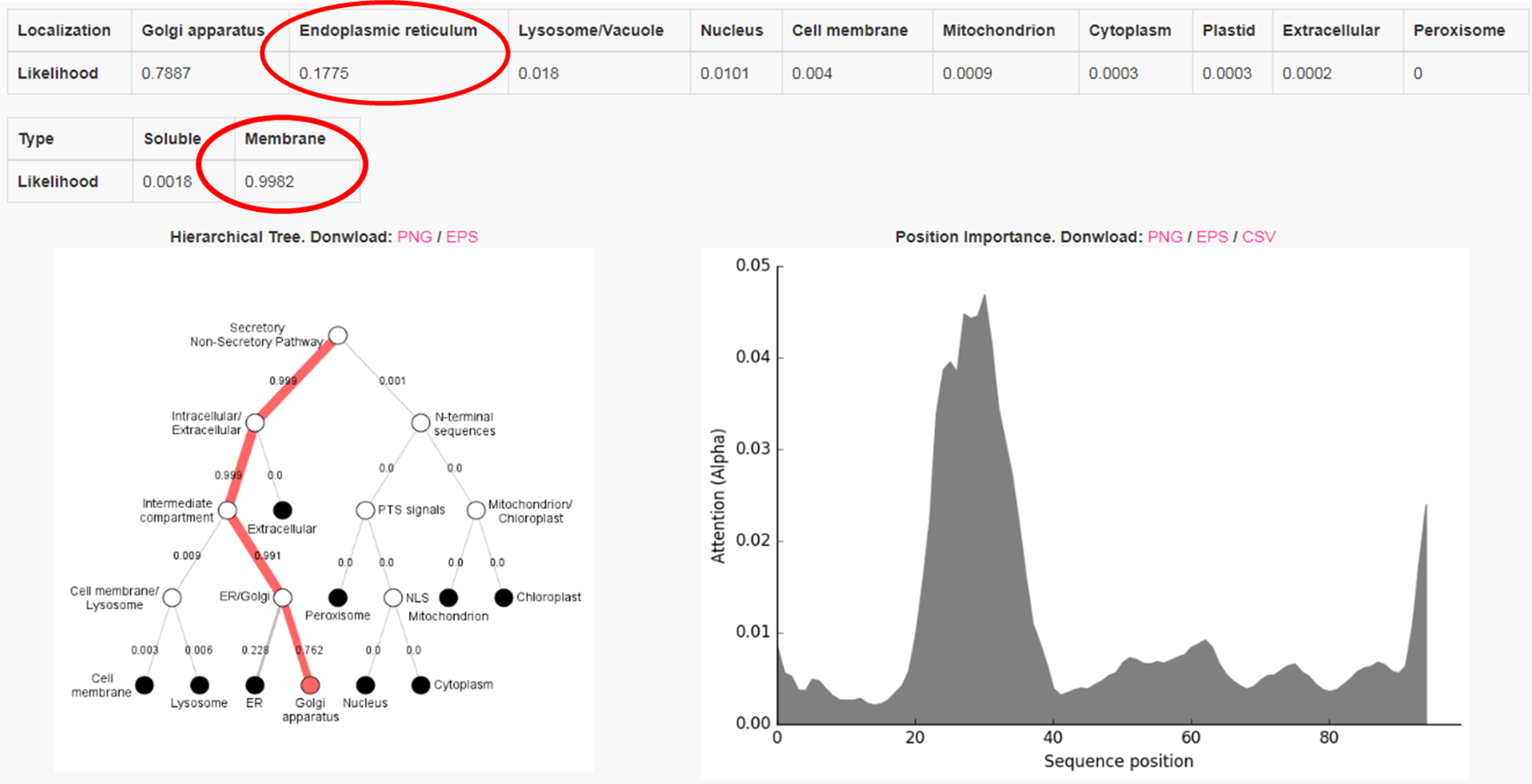

Supplement: Supplementary file 3 — Additional file 3: Figure S2. Prediction of subcellular localization of tick selenoprotein K (XP_002403087.1) by DeepLoc-1.0 algorithm which predicts its localization in the ER/Golgi membrane. [file 13071_2019_3548_MOESM3_ESM.tif]
